# Effects of strict COVID-19 lockdown on patients with obsessive-compulsive disorder compared to a clinical and a nonclinical sample

**DOI:** 10.1192/j.eurpsy.2023.2416

**Published:** 2023-06-01

**Authors:** Giordano D’Urso, Alfonso Magliacano, Bernardo Dell’Osso, Hekla Lamberti, Adalgisa Luciani, Teresa S. Mariniello, Mattia V. Pomes, Lorenza M. Rifici, The COVID in OCD Study Group, Felice Iasevoli, Andrea de Bartolomeis

**Affiliations:** 1 Section of Psychiatry, Department of Neuroscience, Reproductive and Odontostomatological Sciences, University of Naples Federico II, Naples, Italy; 2 IRCCS Fondazione Don Carlo Gnocchi ONLUS, Florence, Italy; 3 Department of Biomedical and Clinical Sciences, University of Milan, Milan, Italy; 4 Department of Psychiatry and Behavioral Sciences, Bipolar Disorders Clinic, Stanford University, Stanford, CA, USA; 5 “Aldo Ravelli” Center for Nanotechnology and Neurostimulation, University of Milan, Milan, Italy

**Keywords:** Adjustment disorder, COVID-19, lockdown, obsessive-compulsive disorder

## Abstract

**Background:**

Symptoms of obsessive-compulsive disorder (OCD) have been reported to increase during the COVID-19 lockdowns because of the hygiene requirements related to the pandemic. Patients with adjustment disorder (AD) may, in turn, represent a vulnerable population for identifiable stressors. In this study, we aimed at assessing potential symptoms changes in OCD patients during the lockdown in comparison with AD patients as well as versus healthy controls (HC).

**Methods:**

During the COVID-related lockdown, we enrolled 65 patients and 29 HC. Participants were tested with four clinical rating scales (Yale–Brown obsessive-compulsive scale and Brown Assessment of Beliefs Scale for OCD patients; Beck Depression Inventory-II and State–Trait Anxiety Inventory-Y for each group) that had been also administered just before the Italian lockdown.

**Results:**

Our results showed that during the lockdown: (i) the symptoms of depression and anxiety increased in all groups, but this increase was most pronounced in HC (*p* < 0.001); (ii) OCD symptoms severity did not increase, but the insight worsened (*p* = 0.028); (iii) the proportion of OCD patients showing hygiene-related symptoms increased (*p* = 0.031 for obsessions of contamination), whereas that of patients with checking-related symptoms decreased.

**Conclusions:**

The lockdown-induced psychological distress apparently changed the characteristics and the pattern of OCD symptoms expression but not their overall severity. This evidence confirms the heterogeneity and changing nature of OCD symptoms, strongly depending on the environmental circumstances.

## Introduction

The Coronavirus Disease (COVID-19) is a highly contagious respiratory disease first reported in Wuhan (China), in late 2019. On March 11, 2020, the World Health Organization (WHO) declared pandemic status [1]. To prevent the contagion, WHO has drawn up hygiene-related guidelines including social distancing, mask use, and frequent handwashing, while many governments have instituted lockdown as a preventive measure. Because of the fear of contagion, restrictions, and the quarantine-related psychological distress, the COVID-19 pandemic has had a dramatic impact on the mental health of the general population [2–4]. Fear of infection, frequent handwashing, and cleaning are typical symptoms of obsessive-compulsive disorder (OCD), a mental disorder characterized by the presence of obsessions and/or compulsions. In a great proportion of OCD patients, the obsessions are related to the fear of contamination and the compulsions consist in repeated washing and cleaning, although there are many other types of obsessions and compulsions as, for example, those related to order and symmetry, checking behaviors, hoarding, and rituals of counting or repeating [5, 6].

In the general population, the pandemic-related psychological distress was strongly related to the risk of contagion and to the urge to apply strict preventive health rules. For this reason, patients with OCD could have been affected more than the general population and, probably, more than patients with other mental disorders. However, the scientific literature reported controversial findings at this regard.

On one hand, a recent large-scale online survey on OCD patients reported a worsening of the OCD symptoms in 76% of participants during the pandemic [7], and another study showed that patients with OCD either started to display new types of obsessions and compulsions or showed again past symptoms that were not present anymore prior to the COVID-19 outburst [8, 9]. An increased incidence of symptoms related to contamination was also reported [10].

On the other hand, in a large cohort of OCD patients no difference was found in OCD symptoms prior to versus during the pandemic [11], and another study found no change in OCD symptoms among children and adolescents, though this was conducted in a small sample [12].

The present study aimed at describing the effect of COVID-19-related lockdown on psychiatric symptoms of patients with OCD and at comparing it with that observed in patients with adjustment disorder (AD) and in a nonclinical sample. We selected the patients with AD as a clinical comparator because they are defined as having developed emotional or behavioral symptoms out of proportion to the severity or intensity of an identifiable stressor. We hypothesized that this could imply a greater vulnerability to the psychological impact of the lockdown.

Moreover, this study refers to the special case of the Campania region of Italy. Italy was the first European country to be hit by the COVID-19 pandemic and, consequently, the first to enact an extended large-scale lockdown. Furthermore, the Campania region, in south of Italy, implemented a very strict control over the adherence to the lockdown and the citizens were repeatedly and vehemently appealed by the authorities through the social networks. This was due to a higher risk of the pandemic spread correlated to the high population density and was unique among the Italian regions. We hypothesized that more severe measures could have had a more pronounced effect on psychiatric symptoms.

We aimed at assessing whether during the pandemic-related lockdown: (i) the increase of anxiety and depression symptoms was greater in OCD and AD patients than in healthy controls (HC); (ii) OCD symptoms worsened; (iii) the proportion of OCD patients showing symptoms related to contamination and washing increased.

## Methods

### Participants

Participants’ enrolment and data collection were performed from April 22 to May 18, 2020, respectively the date of the ethics committee approval and of the end of the mandatory lockdown in Italy.

One hundred and fifty-eight patients, 102 of which with OCD and 56 with AD were selected from the clinical records respectively at the OCD outpatients clinic and at the general psychiatry outpatients clinic of the Psychiatry Unit of the University Hospital “Federico II” of Naples, Italy. The diagnosis of OCD and AD had been formulated by the referring clinicians according to the DSM-5 criteria without the help of standardized diagnostic tools.

The inclusion criteria were that they had been (i) visited during the 3 months preceding the pandemic outburst, and (ii) found clinically stable at that time. The only exclusion criterion was the presence of any psychiatric comorbidity. Among the screened patients, 46 (45%) OCD and 19 (34%) AD accepted to participate, while the remaining resulted not willing to participate for personal reasons or not reachable before the end of the lockdown.

A convenience sample of 29 HC was also enrolled by means of word-of-mouth, according to a snowball sampling.

### Ethics

All procedures were in accordance with the Declaration of Helsinki and its later amendments and were approved by the Ethics Committee for Biomedical Activities of the University of Naples Federico II (approval code 152/20 of 22/04/2020).

### Clinical scales and procedure

The assessment consisted of the following four rating scales, administered in reference to two time points, that is, during the 3 months preceding the pandemic (baseline) and during the COVID-19 lockdown (follow-up):

1. The Yale–Brown obsessive-compulsive scale (Y-BOCS), a clinician-administered rating scale, assessing the current and lifetime presence of OCD symptoms and their severity during the week before the evaluation [13].
2. The Brown Assessment of Belief Scale (BABS) is a seven-item clinician-administered semi-structured scale aimed at assessing the degree of conviction and insight that patients have concerning their beliefs [14].
3. The Beck Depression Inventory-II (BDI-II) is a multiple-choice self-report inventory consisting of 21 items that assess the affective, cognitive, and physical symptoms of depression. Each item is rated from 0 to 3 (from the least to the most severe) [15].
4. The State–Trait Anxiety Inventory-Y (STAI-Y) is a commonly used measure of trait and state anxiety. It includes 20 items for assessing trait anxiety and 20 for state anxiety. For this study, we only used the items assessing the state anxiety and not those assessing the trait anxiety [16].

For the OCD and the AD groups, the baseline data were taken from clinical records reporting scores of the above-mentioned scales (STAI-Y and BDI-II for both groups, Y-BOCS and BABS only for the OCD group) administered during the most recent (no further than 3 months earlier) in-person visit, while the follow-up assessment was made during a psychiatric visit through a video-call.

Only for HC, STAI-Y, and BDI-II scores at both time points were collected during the same call with the baseline evaluation being retrospective in nature. Y-BOCS was also administered to six participants enrolled for the HC group with reference to the two time points of the study. These subjects had reported clinically significant OCD symptoms started during the lockdown when informed about the aims of the present study.

### Statistical analyses

Participants’ baseline characteristics (age, sex, education, illness duration, STAI-Y, BDI-II) were compared across the groups (OCD, AD, HC) by means of nonparametric Kruskall–Wallis H, Mann–Whitney *U* test, or by Chi-square test, as appropriate. We also compared the proportion of patients who were treated with benzodiazepines by means of the Chi-square test.

For the OCD group, the scores of the Y-BOCS and BABS were compared across time (baseline, follow-up) by means of the Wilcoxon signed-rank test. We controlled for a possible effect of benzodiazepines by comparing the scores of the Y-BOCS and BABS in patients taking versus not taking benzodiazepines.

Thereafter, we divided the items of the symptom checklist into the following five different categories, based on the content of obsessions and compulsion: “hygiene,” “checking,” “hoarding,” “symmetry,” and “miscellaneous.” Considering the possible heterogeneity of OCD symptoms displayed by every single patient, a given patient might be included in one or more of the above-mentioned categories. In each of these categories, the mean total scores of the Y-BOCS and BABS were compared across time (baseline, follow-up) by means of the Wilcoxon signed-rank test. In each category, we controlled for a possible effect of benzodiazepines by comparing the scores of the Y-BOCS and BABS in patients taking versus not taking benzodiazepines.

Then, we compared the frequencies of the five categories across time (baseline, follow-up) by means of a Chi-square test separately for: (i) obsessions and compulsions; (ii) obsessions; and (iii) compulsions.

Furthermore, the STAI-Y and the BDI-II scores at the baseline were compared between groups (OCD vs. AD, OCD vs. HC, AD vs. HC) by means of a nonparametric Mann–Whitney *U* test. The same analyses across groups were performed on the scores of the STAI-Y and BDI-II at the follow-up evaluation. Moreover, the scores of the STAI-Y and BDI-II were compared across time (baseline, follow-up) in each group by means of the Wilcoxon signed-rank test. The same analysis was also performed for each OCD symptom category. We controlled for a possible effect of benzodiazepines on STAI-Y and BDI-II scores by comparing their scores between patients taking versus not taking benzodiazepines.

The level of significance was set at 0.05. All analyses were performed using IBM SPSS v.25 (IBM Corp., Armonk, NY).

## Results

### Participants’ characteristics

We enrolled an overall convenience sample of 94 participants (51 females; mean age = 42 ± 15.5 years). Most of the included participants (*n* = 43; 45.7%) had a high school degree, whereas only 2% (*n* = 2) had a primary school degree, and the 27.6% (*n* = 26) and 24.5% (*n* = 23) had a middle school or bachelor/master degree, respectively.

The overall sample included 46 patients with OCD, 19 patients with AD, and 29. The three groups did not differ in terms of age (*H* = 5.26; *df* = 2; *p* = 0.07), sex (*χ* ^2^ = 3.73; *df* = 2; *p* = 0.15), and level of education (*χ* ^2^ = 5.78; *df* = 6; *p* = 0.44; see Table 1 for participants’ characteristics as a function of the study group). Participants with OCD and AD did not differ in their illness duration (*U* = 391.5; *p* = 0.511; Table 1).

**Table 1.** Baseline descriptive statistics of the participants’ as a function of the study group

	OCD (*n* = 46)	AD (*n* = 19)	HC (*n* = 29)	*p*
Sex (M/F)	24/22	5/14	14/15	0.155
Age (years)	39.6 ± 14.8	49.4 ± 16.5	41.0 ± 15.1	0.072
Education (primary/middle/high/bachelor-master)	1/10/21/14	1/8/8/2	0/8/14/7	0.447
Illness duration (months)	200.5 ± 163.8	151.8 ± 100.6	/	0.511
Y-BOCS	17.3 ± 12.5	/	/	**/**
BABS	5.5 ± 6.7	/	/	**/**
STAI-Y	47.9 ± 14.3^a^	41.6 ± 13.2	36.9 ± 11.2^a^	**0.004**
BDI-II	14.8 ± 13.4^a^	11.7 ± 9.9^b^	4.8 ± 4.9^a,b^	**<0.001**

*Note:* Descriptive data are reported as mean ± standard deviation for continuous variables and as counts for categorical variables. Univariate statistics are based upon the Kruskall–Wallis *H* test, Mann–Whitney *U* test, or *χ* ^2^ test, as appropriate. Significant differences across the three groups are reported in bold. The superscript letters a and b indicate significant differences between the two groups.

Abbreviations: AD, adjustment disorder; BABS, Brown assessment of beliefs scale; BDI-II, beck depression inventory-II; F, female; HC, healthy controls; M, male; OCD, obsessive-compulsive disorder; STAI-Y, state–trait anxiety inventory-Y; Y-BOCS, Yale–Brown obsessive compulsive scale.

Family history for the same psychiatric disorder was present in 9 (20%) OCD and in 6 (31%) AD patients; 5 (11%) OCD and 2 (10%) AD patients had previous hospitalizations. The stressors identified for AD patients were familiar or relational issues (*n* = 7; 37%), grief (*n* = 4; 21%), and multiple stressors (*n* = 8; 42%). Patients with OCD were treated with various combinations of benzodiazepines (*n* = 36; 78%), SRIs (*n* = 43; 93%), antidepressants other than SRIs (*n* = 3; 7%), antipsychotics (*n* = 10; 22%), antiepileptics (*n* = 7; 15%), and lithium (*n* = 3; 7%). Patients with AD were treated with benzodiazepines (*n* = 12; 63%), SRIs (*n* = 14; 74%) or antidepressants other than SRIs (*n* = 6; 32%), antipsychotics (*n* = 2; 10%), antiepileptics (*n* = 4; 21%) and lithium (*n* = 1; 5%). The proportion of patients who were treated with benzodiazepines did not differ across the two patient groups (*χ* ^2^ = 1.58; *df* = 1; *p* = 0.208).

Medical comorbidities were more frequent in patients with OCD (14/46, 30.4%) and AD (15/19, 78.9%) than in HC (5/28, 17.8%; *χ* ^2^ = 19.68; *df* = 2; *p* < 0.001), with a significant difference also between OCD and the AD group (*χ* ^2^ = 12.80; *df* = 1; *p* < 0.001). The percentage of individuals that contracted the COVID-19 infection did not differ across groups (OCD = 8/46, 17.3%; AD = 4/19, 21.0%; HC = 3/28, 10.7%; *χ* ^2^ = 1.00; *df* = 2; *p* = 0.606), as well as the percentage of individuals that worked as health professional (OCD = 2/46, 4.3%; AD = 1/19, 5.2%; HC = 1/28, 3.7%; *χ* ^2^ = 0.079; *df* = 2; *p* = 0.961) and the percentage of individuals that were affected economically by the pandemic (OCD = 3/45, 6.6%; AD = 1/18, 5.5%; HC = 2/28, 7.1%; *χ* ^2^ = 0.046; *df* = 2; *p* = 0.977).

### Anxiety and depression

In the overall sample, the mean baseline total score of the STAY-Y was 43.2 (±13.9), whereas the mean baseline total score of the BDI-II was 11.1 (±11.5).

The total score of the STAI-Y (*H* = 11.16; *df* = 2; *p* = 0.004) and of the BDI-II (*H* = 15.23; *df* = 2; *p* < 0.001) differed significantly across the three groups. In particular, the OCD group showed significantly higher STAI-Y and BDI-II scores than HC (*U* = 372.0; *p* = 0.001 and *U* = 310.0; *p* < 0.001, respectively), but not than AD group (*U* = 347.5; *p* = 0.196 and *U* = 395.5; *p* = 0.549, respectively), irrespective from the use of benzodiazepines (all *p* > 0.05).

The AD and the HC group differed significantly in the BDI-II score (*U* = 163.5; *p* = 0.018) but not in the STAI-Y (*U* = 186.5; *p* = 0.060) scores. Descriptive statistics are reported in Table 1.

At the follow-up, the total score the STAI-Y (*H* = 6.74; *df* = 2; *p* = 0.034) and of the BDI-II (*H* = 17.94; *df* = 2; *p* < 0.001) differed significantly across the three groups. In particular, the OCD group showed significantly higher STAI-Y and BDI-II scores than HC (*U* = 440.5; *p* = 0.014 and *U* = 277.5; *p* < 0.001, respectively) but not than AD group (*U* = 345.5; *p* = 0.187 and *U* = 372.5; *p* = 0.352, respectively), irrespective from the use of benzodiazepines (all *p* > 0.05). The AD and the HC group differed significantly in the BDI-II score (*U* = 162.0; *p* = 0.017) but not in the STAI-Y (*U* = 219.5; *p* = 0.237) scores.

**Figure 1.**
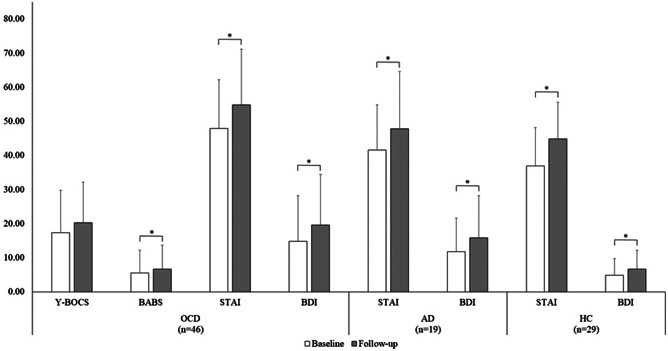
Mean scores of the clinical scales as a function of the time point in each group. Error bars display standard deviation. * indicates significant differences based upon the Wilcoxon signed-rank test at *p* < 0.05. AD, adjustment disorder; BABS, Brown assessment of beliefs scale; BDI-II, beck depression inventory; HC, healthy Subjects; OCD, obsessive-compulsive disorder; STAI-Y, state–trait anxiety inventory-form Y; Y-BOCS, Yale–Brown obsessive compulsive scale.

**Figure 2.**
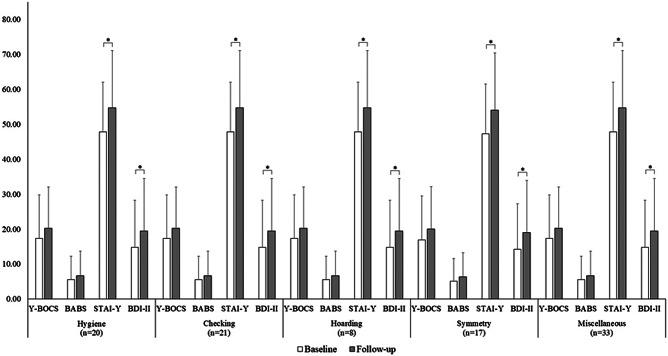
Mean scores of the clinical scales of the OCD group as a function of the time point and of the OCD symptom categories. Error bars display standard deviation. * indicates significant differences based upon the Wilcoxon signed-rank test at *p* < 0.05. BABS, Brown assessment of beliefs scale; BDI-II, beck depression inventory-II; STAI-Y, state–trait anxiety inventory-form Y; Y-BOCS, Yale–Brown obsessive compulsive scale.

In the overall sample, the STAI-Y (*Z* = −5.19; *p* < 0.001) and BDI-II (*Z* = −4.90; *p* < 0.001) scores were significantly higher (+14.1% and 25.1%, respectively) at the follow-up (mean STAI-*Y* = 50.29 ± 14.44; mean BDI-II = 14.81 ± 13.37) compared to the baseline (mean STAI-*Y* = 43.21 ± 13.92; mean BDI-II = 11.10 ± 11.52). In particular, the STAI-Y (OCD: +14.4%; *Z* = −3.23; *p* = 0.001; AD: +14.8%; *Z* = −2.84; *p* = 0.005; HC: +21.6%; *Z* = −3.20; *p* = 0.001) and the BDI-II (OCD: +31.9%; *Z* = −2.96; *p* = 0.003; AD: +34.5%; *Z* = −3.18; *p* = 0.001; HC: +39.7%; *Z* = −2.89; *p* = 0.004) scores increased significantly in all groups (Figure 1). However, no significant changes in both the STAI-Y and the BDI-II were observed between the baseline and the follow-up in patients who did not take benzodiazepines (all *p* > 0.05). Within the single OCD symptom categories, the STAI-Y and the BDI-II scores also increased significantly across time in all categories (all *p* < 0.05; Figure 2), except in patients who did not take benzodiazepines (all *p* > 0.05).

### OCD symptoms

Within the OCD group, the increase of Y-BOCS scores at follow-up compared to baseline only approached statistical significance (mean Y-BOCS = 19.90 ± 13.10 vs. 18.48 ± 12.29; *Z* = −1.95; *p* = 0.05), while the BABS scores increase was significant (mean BABS = 3.34 ± 5.94 vs. 2.90 ± 5.63; *Z* = −2.19; *p* = 0.028; Figure 1). However, in patients who were treated with benzodiazepines the Y-BOCS (mean Y-BOCS = 20.03 ± 12.84 vs. 15.47 ± 13.03; *Z* = −2.58; *p* = 0.01) and the BABS (mean BABS = 6.19 ± 7.19 vs. 4.75 ± 6.57; *Z* = −2.19; *p* = 0.028) scores significantly increased at the follow-up with respect to the baseline, whereas no significant differences were observed in patients who did not use benzodiazepines (all *p* > 0.05).

The Y-BOCS and the BABS scores did not change significantly across time in any of the O-C dimension category (all *p* > 0.05), although the difference in the Y-BOCS score approached significance in the “hygiene” category (*n* = 29; *Z* = −1.77; *p* = 0.07), and the difference in the BABS score approached significance in the “checking” (*n* = 38; *Z* = −1.96; *p* = 0.05) and in the “miscellaneous” (*n* = 41; *Z* = −1.96; *p* = 0.05) categories (Figure 2). However, a significant increase in the Y-BOCS score was observed for the “hygiene” (*n* = 21; *Z* = −2.24; *p* = 0.025), “checking” (*n* = 29; *Z* = −2.11; *p* = 0.035), and “miscellaneous” (*n* = 31; *Z* = −2.18; *p* = 0.029) categories in patients treated with benzodiazepines.

**Figure 3.**
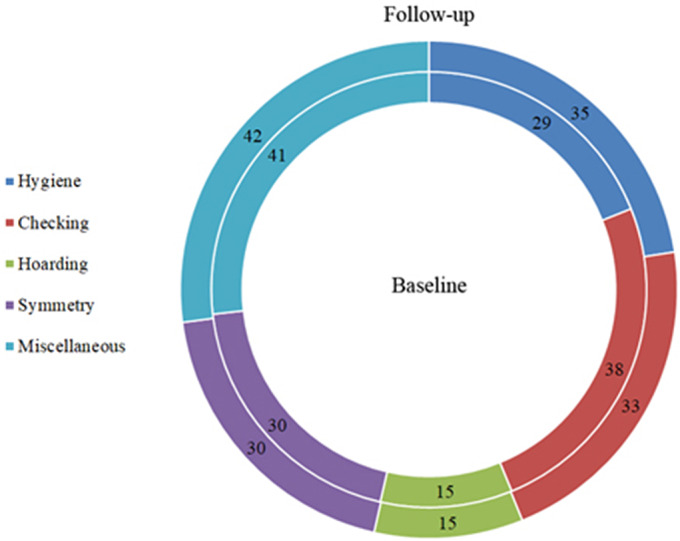
Frequencies of OCD patients in each symptom category at the baseline (inner circle) and at the follow-up (outer circle).

Notwithstanding the frequencies of OCD patients across time increased substantially in the “hygiene” category (baseline = 29 vs. follow-up = 35; +20.69%) and decreased in the “checking” category (baseline = 38 vs. follow-up = 33; −13.16%), no significant differences across time were observed in the number of patients in each category of symptoms (all *p* > 0.05; Figure 3).

**Figure 4.**
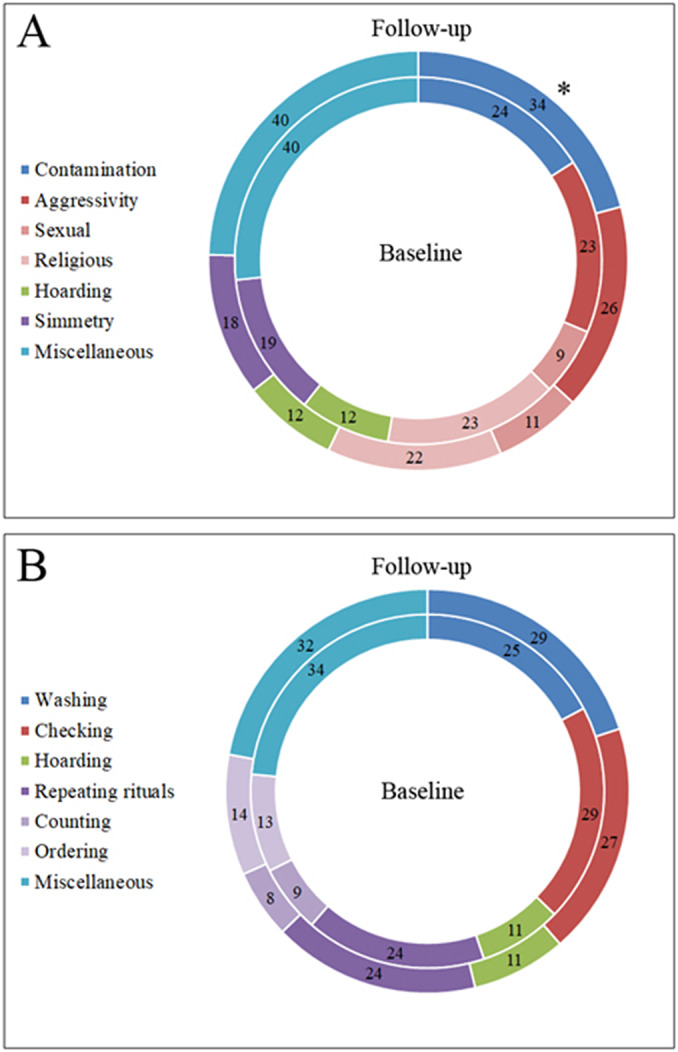
(A) Frequencies of OCD patients for each type of obsessions at the baseline (inner circle) and at the follow-up (outer circle); the frequency of the obsessions related to contamination was significantly higher at the follow-up compared to the baseline (*p = 0.031). (B) Frequencies of OCD patients for each type of compulsions at the baseline (inner circle) and at the follow-up (outer circle).

As regard to the types of obsessions, we found a significant difference across time of the obsession related to contamination (*χ* ^2^ = 4.66; *df* = 1; *p* = 0.031), due to a higher percentage of OCD patients who exhibited this type of obsession at the follow-up (*n* = 34; 73.9%) with respect to baseline (*n* = 24; 52.1%). Within the single OCD categories, the frequency of the obsession related to contamination significantly increased in the “checking” category only (baseline = 23/38, 60.5% vs. follow-up = 31/38, 81.6%; *χ* ^2^ = 4.09; *df* = 1; *p* = 0.043). No other type of obsession showed significant differences across time in any category (all *p* > 0.05; Figure 4A).

As regard to the types of compulsions, no significant differences across time were observed either in the whole OCD group and in any category (all *p* > 0.05; Figure 4B).

The six HC who reported clinically significant OCD symptoms started during the lockdown were not included in the above statistics, that concern the patients already diagnosed with OCD before the pandemic. When the results of the OCD scales of the lockdown-onset patients were included in the statistics, the increase of the total Y-BOCS scores became significant for the hoarding category sample (*p* < 0.05).

## Discussion

The present study aimed at analyzing the impact of severe pandemic-related restrictions on the psychiatric symptoms of patients with OCD, and at comparing this impact with that observed in two control groups, that is, AD patients and HC. In all groups, we explored the levels of anxiety (STAI-Y) and depression (BDI-II), while only in OCD patients we assessed the obsessions and compulsions severity (Y-BOCS), and the levels of insight/delusionality related to the OCD symptoms (BABS). Finally, besides the overall severity of OCD symptoms, we aimed at detecting their possible qualitative changes by assessing the proportion of OCD patients that displayed each symptom subtype at the two time points.

We found that all groups showed significantly higher levels of anxiety and depression during the lockdown compared to baseline. This result is in accordance with previous studies showing higher levels of emotional distress, anxiety, and depression symptoms during the COVID-19 pandemic in the general population [17], as well as among patients with preexisting psychiatric disorders [18–20]. Contrary to our expectations, in our sample the greatest increase in anxiety and depression symptoms was observed in the HC group. Considering that the OCD and the AD groups had higher score of these symptoms compared to HC both at baseline and at follow-up, the smaller increase of the psychiatric patients might be due to a ceiling effect. Moreover, we can speculate that the everyday life was affected more in HC than in subjects already suffering from a psychiatric condition. Looking at the OCD group more into details, we observed no differences in the increase of anxiety and depression symptoms among the five categories of patients obtained according to the specific OC symptoms displayed, indicating that anxiety and depression increased independently from the OC subtype. As a matter of fact, considering the significant increase of depression and anxiety in all groups, new methods for detecting demographic and clinical indicators of vulnerability to the onset and/or worsening of such widespread symptoms in case of severe restrictions could help preventing much of the suffering related to such dramatic contingencies [21].

Regarding the severity of OCD symptoms, we did not detect a significant increase over time, neither in the OCD group considered as a whole, nor after dividing it according to the single categories of OCD symptoms. This result is also in contrast with previous evidence. In fact, during the outbreaks of other infectious diseases, such as the Severe Acute Respiratory Syndrome, the Middle East Respiratory Syndrome, and the Influenza, a worsening of OCD symptoms severity was reported [22]. In the case of COVID-19 pandemic, greater OCD symptoms have been detected by several studies [7, 10, 23]. There might be several different reasons for this discrepancy, among which major methodological differences. Firstly, previous studies [7, 23] enrolled hundreds of OCD patients, while we had only 46, which probably prevented us to reach the statistical significance. Moreover, those two studies do not refer specifically to the condition of stay-at-home restriction, but more generally to the pandemic. Another possible source of variability among studies is the geographic area where the patients were recruited. Our sample is unique in that it is composed of patients living in a region of Italy (Campania) where the authorities implemented a particularly strict and sustained control over the compliance with the restrictions, in consideration of the very high population density (the highest in Europe in certain areas) and the consequent greater risk of infection spread. Moreover, the different rate of nonparticipation to the study due to current COVID-19 symptoms or to not having access to the necessary technology might also have yielded to differences among the various geographic areas. In line with these considerations, also the Van Ameringen’s study [7] reported geographic variability in the results, with a decreased risk of OCD symptoms worsening associated with being from Europe. In addition, the baseline severity of OC symptoms might have played a role, since Prestia et al. [10] showed that the extent of worsening was inversely correlated to the prelockdown severity of OC symptoms. As a matter of fact, the average Y-BOCS score of the Prestia’s sample at baseline was considerably lower than ours (15.97 vs. 18.48). Finally, the study by Van Ameringen et al. adopted a very different assessment method, that is, an online survey whose link was posted to the sites of social media, OCD clinics, and OCD research centers. During the survey, the patients were asked to complete several psychiatric rating scales, among which the obsessive-compulsive inventory-revised (OCI-R) assessing the OCD symptoms over the last month. For the symptoms change, the authors did not rely on a comparison between two different clinical evaluations performed by health professionals, as we did, but on the patients’ perception, reported during one single online survey. This might have led to an overestimation of the worsening in their study, so partially accounting for the difference in the detected increase compared to ours. Another possible explanation is that we included only patients already in treatment and clinically stable before the lockdown and this could have had a protective effect against symptoms worsening.

Unlike the Y-BOCS scores, another measure of OCD severity increased significantly at the follow-up, that is, the BABS, which revealed lower levels of insight of OCD symptoms. We speculate that this was because OCD patients felt more justified and in accordance with social expectations when performing their OCD rituals during the pandemic, considering the global urge to keep social distance, wash hands, wear masks, clean and disinfect surfaces to prevent the infection’s spread [24]. This probably led to a reduced ego-dystonia and discomfort with those contamination fears that were already present before the pandemic, but finally appeared more realistic during the lockdown. Moreover, if combining this evidence with the above-discussed nonincrease of symptom severity at the Y-BOCS, we can also speculate that the reduction of the feeling of shame that usually accompanies the insight of OCD symptoms might have paradoxically protected patients from the worsening of symptoms.

In line with our expectations, the distribution of the different subtypes of OCD symptoms in the OCD sample changed during the lockdown, with an increased rate of patients showing hygiene-related symptoms. The pathological nature of the hygiene-related behaviors of OCD patients was distinguishable from the pandemic-related increased attention to hygiene of the general population by the extent of these behaviors both in quantitative (e.g., duration and repetition of behaviors beyond what recommended and/or reasonable) and qualitative (e.g., cleaning of surface not exposed to the possibility of contamination) terms as well as by the interference on daily functioning. More specifically, we found a significant increase in the percentage of patients with contamination-related obsessions at follow-up (73.9%) compared to baseline (52.1%). This evidence is consistent with the hypothesis that the content of OCD symptoms is not random [25] and, more specifically, can change depending on the circumstances [26]. Interestingly, parallel to the increase of hygiene-related symptoms, we observed a reduced rate of checking symptoms, while the other three symptom categories remained unvaried. Moreover, in the “checking” group of patients, the frequency of contamination-related obsessions increased significantly at the follow-up, and no similar findings were detected in the other categories. A possible explanation for this is that hygiene-related and checking symptoms have in common some specific pathophysiological features, and perhaps represent a distinct endophenotype in the context of the OCD spectrum. This would account for a higher probability of transformation of the symptoms of one category into those of the other.

As a serendipitous finding, we found that six subjects screened for the HC group had developed clinically relevant OCD symptoms during the lockdown. When their Y-BOCS scores were included in the statistics of the OCD group, the mean increase at follow-up became significant for the hoarding category sample. This evidence points to the multifactorial nature of OCD and to the fact that predisposed individuals can develop clinically relevant OCD symptoms when subject to stressors that are beyond their individual tolerance threshold. Moreover, the specific category of symptoms displayed by the lockdown-onset OCD patients suggests that the uncertainty about the future and the shortage of essential goods and items had probably triggered the hoarding behaviors. However, once arisen, these behaviors were not limited to the essential goods, but extended to unnecessary possessions, which makes them pathological in nature. Another possible explanation of the onset of hoarding symptoms in previously healthy individuals is the social isolation imposed by the lockdown, which could have led predisposed subjects to anthropomorphize nonhuman entities, as is commonly observed in clinical samples of hoarders [27]. Finally, the possible specific vulnerability of these six subjects to develop hoarding-related symptoms gives support to the DSM-5 choice to consider the hoarding disorder a distinct nosographical entity separated from OCD.

It is also worth noting that the use of benzodiazepines influenced the results. Specifically, only the patients taking drugs from this class showed symptoms worsening at the follow-up. A possible explanation of this finding is that taking benzodiazepines might be a proxy for a higher psychopathological vulnerability.

In summary, the main findings of this study consist in the evidence that under a particularly strict pandemic-related condition of restriction: (i) obsessive-compulsive symptoms of OCD patients do not change in severity but in some of their qualitative characteristics, that is, checking behaviors and insight reduce while hygiene-related symptoms increase; (ii) Anxiety and depression symptoms increase more in HC than in AD and OCD patients; and (iii) New onset OCD patients mainly display hoarding symptoms.

To our knowledge, this is the first study on the matter where an objective and in-person assessment of prepandemic psychopathological symptoms is compared with an assessment performed with the same rating scales during the lockdown. Moreover, we enrolled our sample from a geographical context (Campania region) which is probably unique for the particularly severe control exerted by the authorities over the compliance with the sanitary provisions. This could have led to an exceptional perspective on the course of the symptoms considered in these populations.

From a practical point of view, the evidence that our hypotheses have been largely falsified gives support to the need of more accurate algorithms for the assessment of psychopathological risk in conditions of emergency [28].

However, this study has some methodological flaws. First, the psychiatric diagnosis in the clinical samples had been formulated by the referring clinicians without the help of standardized diagnostic tools and was based only on their clinical judgment according to DSM-5 criteria. Second, the small sample size might have prevented us from detecting relevant differences at the follow-up, among which OCD symptoms worsening. In fact, the short interval of time between the date of our ethics committee’s approval and that of the end of the mandatory lockdown in Italy was the cause for the reduced dimensions of the sample. In this short time, and in the unusual situation of the lockdown, a considerable proportion of patients selected from their clinical records resulted not reachable, or unwilling to participate for personal or health reasons, accounting also for the low recruitment rate of the study (45% for the OCD group, 34% for the AD group). Moreover, our choice not to extend the enrolment to patients visited beyond 3 months before the pandemic also limited the sample. This choice was due to the confounding effect of possible life events occurred between a more distant time point and the lockdown. The narrow time window for the enrolment was also responsible for the heterogeneity in the sample size of the three study groups. Even though we used nonparametric statistical tests, the size difference between the three samples might have influenced the findings. Third, we only included patients from the Campania region of Italy, which hampers the generalizability of the results. Finally, we adopted heterogeneous procedures (i.e., clinical records vs. video-calls) for collecting baseline data in psychiatric patients and HC. This difference probably jeopardized the comparison of baseline findings in the three groups. Moreover, for HC, baseline and follow-up data were collected within a single video call during the lockdown. This might have affected baseline data reliability due to physiological oblivion, state-dependent memory recall, and selective attention biases of respondents.

## Conclusions

To our knowledge, this is the first study that investigated the effect of COVID-19-related lockdown on psychiatric symptoms of patients with OCD, compared to AD and HC, in an Italian population subjected to particularly strict provisions against the COVID-19 pandemic spread.

Besides confirming previous evidence of higher incidence of psychopathology during lockdown, our study points to the pleomorphic and changing nature of OCD, whose manifestations are distributed on a continuum between normality and pathology and are tightly related to environmental circumstances. The evidence gathered gives support to the need of more accurate algorithms for the assessment of psychopathological risk in conditions of emergency.

## Data Availability

The data that support the findings of this study are available from the corresponding author, G.D., upon request.
